# Delay in the diagnosis and treatment of breast cancer in Vietnam

**DOI:** 10.1002/cam4.4244

**Published:** 2021-10-19

**Authors:** Sang M. Nguyen, Quang T. Nguyen, Lan M. Nguyen, Anh T. Pham, Hung N. Luu, Huong T. T. Tran, Thuan V. Tran, Xiao‐Ou Shu

**Affiliations:** ^1^ Division of Epidemiology Department of Medicine Vanderbilt Epidemiology Center Vanderbilt‐Ingram Cancer Center Vanderbilt University School of Medicine Nashville Tennessee USA; ^2^ National Cancer Hospital Vietnam National Cancer Institute Hanoi Vietnam; ^3^ Hanoi Medical University Hanoi Vietnam; ^4^ Hanoi Oncology Hospital Hanoi Vietnam; ^5^ UPMC Hillman Cancer Center University of Pittsburgh Medical Center Pittsburgh Pennsylvania USA; ^6^ Department of Epidemiology Graduate School of Public Health University of Pittsburgh Pittsburgh Pennsylvania USA; ^7^ Ministry of Health Hanoi Vietnam

**Keywords:** breast cancer, delay in diagnosis and treatment, delay time, patient barriers, Vietnam

## Abstract

**Background:**

Delays in diagnosis and treatment from first noticeable breast cancer symptoms are associated with poor outcomes. Understanding the reasons and barriers for patients’ delay in seeking medical care is critical to mitigating the problem.

**Methods:**

In‐person surveys were conducted among 462 women, aged 18–79, with incident breast cancer cases, recruited from two cancer hospitals in North Vietnam. Delay, defined as the time interval between symptom recognition to the diagnosis and initiation of treatment equal to or exceeding 3 months, was categorized as follows: no delay (<3 months), moderate delay (3–8 months), and serious delay (≥9 months). Multivariable multinomial logistic regression was applied in data analyses.

**Results:**

Over one‐quarter patients (31.5%) experienced moderate delays, and close to one‐fifth (17.5%) experienced serious delays. Adjusted odds ratios and 95% confidence intervals for moderate and serious delays were 5.60 (3.00–10.47) and 4.25 (2.05–8.85) for financial and physical barriers, respectively. Moderate delay was positively associated with psychological barriers (5.55 [1.75–17.57]) and lack of proper knowledge (3.15 [1.47–6.74]). The associations of barriers with delays in diagnosis and treatment appeared stronger among women living in rural areas. A lack of proper knowledge was significantly associated with delay among young women (<45 years old) and those with high incomes, while psychological barriers were significantly associated with delay among older women (≥45 years old).

**Conclusion:**

Delays in diagnosis and treatment are common among Vietnamese breast cancer patients and are affected by several noted barriers. Proper policy needs to be developed to address this public health issue.

## Graphical abstract

A case cohort study found that delay in diagnosis and treatment of breast cancer is common among Vietnamese women and is affected by financial and physical barriers, psychological barriers, and lack of proper knowledge. Proper policy needs to be developed accordingly to address this public health issue.

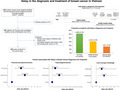

## 1 INTRODUCTION

Breast cancer is one of the most common cancers and the leading cause of cancer death in women, with approximately 2.3 million incident cases worldwide in 2020.^1^ The breast cancer incidence rate is low in Vietnam, a low‐ and middle‐income country (LMIC), compared with the United States (U.S.) and other high‐income countries (HICs), but has increased rapidly over the last decades. This is in accordance with trends in nations where populations have decreased fertility rates and increased overweight and obesity prevalence, as well as improvements in breast cancer screening and awareness.^2^, ^3^ In 2020, the International Agency for Research on Cancer reported an estimated 21,555 new breast cancer incident cases and 9345 deaths in Vietnam, making breast cancer the most common cancer and fourth leading cause of cancer‐related death among Vietnamese women.^1^

A high proportion of breast cancer patients in LMICs are diagnosed at late stages, which results in low efficacy and greater financial burden of cancer treatment and places women at a higher breast cancer mortality risk.^4^, ^5^ A systematic review of 87 breast cancer studies of nearly 102,000 patients showed that a delay of at least 3 months between the onset of breast cancer symptoms and initiation of treatment was an important determinant for late‐stage presentation and poor survival.^6^ Breast cancer patients in LMICs may endure symptoms for up to 8–12 months before diagnosis and treatment.^7^ A delay in diagnosis and treatment, often referred to as total delay time, has been defined as a delay exceeding 3 months from symptom recognition to the initiation of treatment, and is typically divided into patient delay time (i.e., a delay in seeking medical care from the first self‐discovery of symptom onset to the first medical visit) and health system delay time (i.e., a delay within the health care system from the first medical visit to the initiation of cancer treatment).^8^ Patient delay time may account for a greater proportion of total delay time.^9^ Women in LMICs may experience longer patient delay times due to having poor knowledge about breast cancer and barriers but are able to be diagnosed and treated upon seeking medical care.^10^ Therefore, understanding factors for patients’ delay in seeking medical care is critical for the development of effective interventions in order to minimize total delay time and improve prognosis.

To our knowledge, no epidemiological study has been conducted in Vietnamese women to systematically evaluate underlying reasons and barriers for patients’ delay in seeking medical care for breast cancer. To fill the knowledge gap, we conducted a case‐only analysis of breast cancer patients enrolled in the Vietnamese Breast Cancer Study (VBCS) to evaluate the extent of a delay from the first symptom recognition to the initiation of diagnosis and treatment and to identify reasons and barriers related to the delay.

## 2 METHODS

### 2.1 Study population

The VBCS included 501 women between 18 and 79 years old with newly diagnosed breast cancer (clinical diagnosis). Patients were recruited from inpatient surgical units and chemotherapy inpatient and outpatient units of two major cancer hospitals in North Vietnam, the Vietnam National Cancer Hospital and Hanoi Oncology Hospital, from July 2017 to June 2018 (response rate of 93.1%). We excluded patients if they had a history of any cancer prior to the diagnosis of breast cancer. Written, informed consent was obtained from all VBCS participants. Approvals for human subject research were obtained from the Vietnam National Cancer Institute and Vanderbilt University Medical Center.

We excluded participants who were subsequently confirmed to have a benign tumor, based on the pathological review (*n* = 9) and who self‐reported as non‐symptomatic breast cancer (*n* = 10). Participants with incomplete medical chart reviews from December 2019 to May 2020 were also excluded (*n* = 20). Finally, 462 cases remained in the current study (Figure S1).

In‐person interviews were conducted through the Research Electronic Data Capture (REDCap) mobile application at study enrollment by trained interviewers using a structured questionnaire, which collected information on patients’ demographics, socioeconomic characteristics, lifestyle factors, family history of cancer, breast cancer‐related symptoms and dates of notice, and reasons for a delay in seeking medical care. Additionally, clinical features were abstracted by reviewing patients’ medical records, which were collected and directly entered into the REDCap data management platform hosted at Vanderbilt University.^11^

### 2.2 Measurement of delay in diagnosis and treatment

Total delay time, the length of the time interval from the first signs/noticeable breast cancer symptoms to the diagnosis and initiation of treatment at these two hospitals was calculated. The 15th day of the month was used as a proxy in a case in which the month and year were known, but the exact date of the first signs or noticed symptoms was not documented. Based on the literature, we defined no delay in diagnosis and treatment as <3 months of total delay time.^8^, ^12^ A moderate delay was defined as a delay from 3 to 8 months, and a serious delay was defined as a delay of at least 9 months.

### 2.3 Reasons and barriers for the delay in seeking medical care

Twelve self‐reported reasons related to a delay in seeing a doctor upon first noticed breast cancer‐related symptoms were assessed and grouped into three major categories of barriers, that is, financial and physical barriers, psychological barriers, and a lack of proper knowledge. Financial and physical barriers were combined to include four reasons: too expensive, too busy (need time away from family/work), hospital(s) too far away (distance/transportation), and too sick to travel. Psychological barriers (including barriers of behaviors and beliefs) were identified as a combination of four reasons: fear of doctor/hospital, fear of losing a job, sought herbal or other alternative medical treatment, and confidential concerns. A lack of proper knowledge was interpreted as patients being unconcerned about their symptoms. Due to no patients reporting a fear of discrimination or religious reasons, we excluded these two reasons from our analysis.

### 2.4 Statistical analysis

Descriptive statistics of patients’ demographic characteristics were computed in percentages for categorical variables and mean and standard deviation, or median, and interquartile range (IQR) for continuous variables. Associations between barriers for delays in seeking medical care and delays in diagnosis and treatment (no delay vs. moderate and serious delays) were evaluated using multinomial logistic regression models. Odds ratios (ORs) and 95% confidence intervals (CIs) were calculated with adjustments for potential confounders. Potential confounders adjusted in the analyses were age at diagnosis, education levels, average annual per capita income, residence, and the number of noticed symptoms. We performed analysis stratified by age at 45 years old or younger, average annual per capita income levels (tertile distributions), and residence (urban area/suburban area vs. rural area). Likelihood ratio tests were used to assess multiplicative interactions between these variables and barriers for delays in seeking medical care. Sensitivity analyses were performed by excluding women who reported first breast cancer detection via mammographic screenings and routine health examinations (*n* = 30) and women for whom there was a delay in diagnosis and treatment of 12 months or longer (*n* = 73). A two‐sided *p*‐value of <0.05 was considered statistically significant. Stata 14.0 software package (StataCorp) was employed for analyses.

## 3 RESULTS

The mean age at the time of diagnosis and treatment of 462 study participants was 49.4 years. Approximately 61.5% of participants lived in rural areas, 48.1% worked in agriculture or industrial and construction, and over 80% were married. Forty‐one percent of cases had attained a high school, college or higher education. Only 3.7% and 17.5% of participants, respectively, reported a family history of breast cancer or a family history of any other cancer among first‐degree relatives (Table 1).

**TABLE 1:** Characteristics of participants (*N* = 462)

Characteristics	*n* (%)
Age at diagnosis (Mean ± SD; years old)	49.5 ± 10.7
<45	147 (31.8)
45–54	175 (37.9)
55–64	111 (24.0)
65+	29 (6.3)
Marital status	
Married	388 (84.0)
Single/separated/divorced/windowed	75 (16.0)
Education	
Never had formal education/primary school	70 (15.2)
Middle school	204 (44.2)
High school	106 (22.9)
College or higher	82 (17.7)
Occupation	
Workers in agriculture/in industrial and construction	222 (48.1)
Governors/managers/officers	99 (21.4)
Servicers/Sellers/homemakers/students and others	141 (30.5)
Average annual per capita income (Mean ± SD; million Vietnamese dong)	22.7 ± 16.2
Location	
Urban/Sub‐urban area	178 (38.5)
Rural area	284 (61.5)
Travel time to a health care setting (min)	
<30	137 (29.6)
30	101 (21.9)
31–60	98 (21.2)
>60	126 (27.3)
Family history	
Breast cancer‐first degree	17 (3.7)
Other cancers‐first degree	81 (17.5)

Abbreviation: SD, standard deviation. VND: Vitaminese currency dong.

Tumor stages T2, N0, and M0 were the most frequent among breast cancer patients. Over half (54.7%) of participants were diagnosed at stage 2A or earlier, while 22.8% of participants were diagnosed at stage 3 or later. For molecular subtype, most participants had luminal B (45.7%) and HER‐2 overexpression (19.9%) (Table S1).

Only 30 breast cancer cases were detected through routine health examinations (5.4%) and mammographic screenings (1.1%). For the remaining 93.4% of cases, breast cancer diagnosis was made after symptoms or lumps were noticed. Common first noticeable symptoms included a breast lump or mass (96.3%), pain or tenderness (31.7%), and change in breast size (24.0%). Approximately 14.3% of patients reported having more than two symptoms (Table 2).

**TABLE 2:** Symptoms, diagnosis modality, barriers/reasons for the delay in seeking a medical care and delay in diagnosis and treatment

	*n* (%)
	*N* = 462
The first noticed symptoms in breast	
Lump or mass	445 (96.3)
Pain or tenderness	144 (31.7)
Nipple discharge	30 (6.5)
Skin ulceration or rash	11 (2.4)
Infection	18 (3.9)
Skin dimpling	18 (3.9)
Change size	111 (24.0)
The number of symptoms noticed	
Only one	250 (54.1)
Two	146 (31.6)
Above two	66 (14.3)
Circumstance of breast cancer detection	
Mammographic screening	5 (1.1)
Routine health examination	25 (5.4)
Self‐noticed systems/lump	431 (93.4)
Visited a doctor right after the first noticed problems with breast	
Yes	340 (73.5)
No	122 (26.4)
	*N* = 122
Barriers and reasons for the delay in seeking a medical care	
Financial and physical barriers	
Too busy (need time away from family/work)	63 (51.6)
Cost/too expensive	19 (15.6)
Hospital(s) is too far away (distance/transportation)	17 (13.9)
Too sick to travel	2 (1.6)
Any of above	79 (64.8)
None of above	43 (35.2)
Psychological barriers	
Sought of herbal or other alternative medicine treatment	13 (10.7)
Confidential concerns	9 (7.4)
Fear of doctor/hospital	8 (6.6)
Fear of losing job	1 (0.8)
Any of above	23 (18.9)
None of above	99 (81.1)
Lack of proper knowledge	
Unconcerned about symptoms	42 (34.4)
	*N* = 462
Total delay time (Median [IQR]; months)	2.4 [1.1–7.1]
Seeking medical care after the first symptom recognition	2.0 [0.9–5.4]
Postponed seeking medical care after the first symptom recognition	5.5 [2.5–9.3]
Delay in diagnosis and treatment	
No delay (< 3 months)	238 (51.5)
Moderate delay (3–8 months)	143 (31.0)
Serious delay (≥9 months)	81 (17.5)

Abbreviation: IQR, interquartile range.

Over 73% of participants reported having seen a doctor upon the first notice of symptoms. Among the remaining patients who did not immediately see a doctor, 64.8% reported having financial and physical barriers, one‐third reported having a lack of proper knowledge, and 18.9% reported psychological barriers.

The overall median total delay time was 2.4 months (IQR: 1.1–7.1 months); 5.5 months (IQR: 2.5–9.3 months) for patients who postponed seeking medical care after first symptom recognition, which was much longer than patients who sought medical care after first symptom recognition (median and [IQR]: 2.0 months [0.9–5.4]). The percentages of breast cancer patients who experienced moderate and serious delays in diagnosis and treatment were 31.0% and 17.5%, respectively (Table 2).

Moderate and serious delay groups tended to be more likely to have lower incomes than the no‐delay group. As expected, they also had more reasons/barriers for delays in seeking medical care than the no‐delay group (Table S2).

Multivariable analyses showed that only women who had financial and physical barriers were more likely to experience both moderate and serious delays. Adjusted ORs and 95% CIs for moderate and serious delays were 5.60 (3.00–10.47) and 4.25 (2.05–8.85) for financial and physical barriers, respectively. Moderate delay was positively associated with psychological barriers (OR = 5.55; 95% CI: 1.75–17.57) and lack of proper knowledge (OR = 3.15 (1.47–6.74) (Table 3).

**TABLE 3:** Adjusted odd ratio for the delay in diagnosis and treatment by barriers for delay in seeking medical care^a^

	Delay in diagnosis and treatment				
	No delay *N* = 238	Moderate delay *N* = 143		Serious delay *N* = 81	
	*n*	*n*	aOR (95% CI)	*n*	aOR (95% CI)
Financial and physical barriers					
No	221	100	1.00	61	1.00
Self‐reported	17	43	5.60 (3.00–10.47)	20	4.25 (2.05–8.85)
Psychological barriers					
No	234	129	1.00	76	1.00
Self‐reported	4	14	5.55 (1.75–17.57)	5	2.93 (0.74–11.7)
Lack of proper knowledge					
No	226	122	1.00	72	1.00
Self‐reported	12	21	3.15 (1.47–6.74)	9	2.50 (0.98–6.38)

Abbreviations: aOR, adjusted odds ratio; CI, confidence interval.

^a^ Multivariable multinomial logistic regression model was adjusted for age at diagnosis, education levels, average annual per capita income, residence and number of symptoms.

Stratified analyses showed that significant associations between financial and physical barriers and moderate or serious delays appeared to be stronger among participants living in rural areas (OR = 9.14; 95% CI: 3.69–22.64) and having middle incomes (OR = 7.55; 95% CI: 2.73–20.93) or high incomes (OR = 6.20; 95% CI: 1.60–23.96) compared with participants living in urban areas and having low incomes. A lack of proper knowledge was significantly associated among individuals aged <45 years old (OR = 10.55; 95% CI: 2.18–51.10), living in rural areas (OR = 3.46; 95% CI: 1.47–8.16) and having high incomes (OR = 7.24; 95% CI: 1.23–42.55), whereas psychological barriers were only significantly associated with moderate or serious delays among individuals aged more than 45 years old (OR = 5.58; 1.56–19.93) and living in rural areas (OR = 6.23; 95% CI: 1.36–28.53). However, most multiplicative interaction tests were not statistically significant, with exceptions of interactions between financial and physical barriers and residence (*p* for interaction = 0.04) and between a lack of proper knowledge and age (*p* for interaction = 0.04) (Table 4).

**TABLE 4:** Adjusted odd ratio for the delay in diagnosis and treatment by barriers for the delay in seeking medical care in stratified analyses^a^

Barriers for the delay in seeking a medical care	Delay in diagnosis and treatment	
	Moderate/serious delay aOR (95% CI)	*p* for interaction
Financial and physical barriers		
Age < 45 years old	6.09 (2.07–17.86)	0.72
Age ≥ 45 years old	5.34 (2.58–11.03)	
Low income	3.95 (1.61–9.68)	0.67
Middle income	7.55 (2.73–20.93)	
High income	6.20 (1.60–23.96)	
Urban/Sub‐urban area	2.93 (1.24–6.91)	0.04
Rural area	9.14 (3.69–22.64)	
Psychological barriers		
Age < 45 years old	1.49 (0.13–17.42)	0.46
Age ≥ 45 years old	5.58 (1.56–19.93)	
Low income	3.95 (0.81–19.23)	0.96
Middle income	6.17 (0.66–57.93)	
High income	4.07 (0.41–40.50)	
Urban/Sub‐urban area	2.86 (0.50–16.28)	0.44
Rural area	6.23 (1.36–28.53)	
Lack of proper knowledge		
Age < 45 years old	10.55 (2.18–51.10)	0.04
Age ≥ 45 years old	1.79 (0.76–4.23)	
Low income	2.40 (0.82–6.99)	0.67
Middle income	2.64 (0.72–9.60)	
High income	7.24 (1.23–42.55)	
Urban/Sub‐urban area	1.92 (0.48–7.61)	0.45
Rural area	3.46 (1.47–8.16)	

Abbreviations: aOR, adjusted odds ratio; CI, confidence interval.

^a^ Multivariable logistic regression model was adjusted for age at diagnosis, education levels, average annual per capita income, residence, number of symptoms, and except for the corresponding variable used for stratification.

Sensitivity analyses excluding 103 participants did not materially change the above‐reported associations (data not shown).

## 4 DISCUSSION

In this study of 462 Vietnamese breast cancer patients, we found substantial delays, including moderate (31.0%) and serious (17.5%) delays in diagnosis and treatment, despite 73.5% of patients who reported seeking medical care soon after the first noticeable symptoms. We also observed that participants, particularly women living in rural areas, who had financial and physical barriers, psychological barriers, and a lack of proper knowledge, were more likely to experience delays in diagnosis and treatment. Furthermore, we found that the lack of proper knowledge and delay association was more pronounced among individuals aged <45 years old, and financial and physical barriers and delay associations were more prominent among rural patients.

Several studies have investigated the impact of the time interval between the onset of breast cancer symptoms and the start of cancer treatment on prognosis and showed that delays in diagnosis and treatment increased the chances of cancer diagnosis at a late stage, which led to more aggressive treatment and poor survival.^6^, ^8^, ^13^ Our study reported a median total delay time of 2.4 months among Vietnamese women with breast cancer, which was generally shorter than the median total delay time reported by other LMICs, but longer than that reported by HICs.^13^, ^14^ A meta‐analysis of 12 European LMICs reported a mean total delay time of 3.6 months (range: 2.9–7.4 months) among 6588 women with breast cancer.^14^ In addition, in our study, we found that 51.5% of breast cancer patients were diagnosed and commenced breast cancer treatment within 3 months from the first noticeable symptoms. A review of total delay time in 10 HICs and 23 LMICs showed that women from HICs had shorter total delay times, ranging from 1.0 to 1.6 months, with more than 60% of breast cancer patients commencing treatment <3 months after the first symptom recognition, whereas women from LMICs experienced longer total delay times, ranging from 5.5 to 8.0 months, with fewer than 30% beginning treatment within 3 months after an abnormal screening or symptom discovery.^13^

Among factors contributable to delay in the diagnosis and treatment of breast cancer, patients’ sociodemographic characteristics and clinical features have been extensively evaluated, but few studies have examined the influences of specific issues such as patients’ reasons or barriers.^13^ Barriers such as “too busy to schedule an appointment with a health care professional” and “financial constraints” were frequently reported, but evidence was primarily derived from qualitative studies.^15^, ^16^ Studies have found that breast cancer patients failed to recognize the seriousness of their symptoms or had no fear of their symptom discovery due to a lack of proper knowledge, which resulted in a delay in seeking care or later‐stage at diagnosis.^15^, ^17^, ^18^ Emotional barriers, including embarrassment or fear related to a worry over diagnostic tests, service barriers such as concerns about wasting doctors’ time, and difficulties with accessing and making appointments, have been explored.^15^, ^16^, ^19^ However, most of these studied were conducted in developed countries and results may not be generalized to LMICs. We only identified one qualitative study (sample size = 18) that highlighted significant barriers for women with breast cancer in Vietnam, including a lack of knowledge, poor communication with health care providers, economic pressures of seeking medical care, and negative emotions and experiences accessing and using breast cancer treatment services.^20^ Our study is the first to investigate associations of patient self‐reported barriers for delays in diagnosis and treatment among Vietnamese breast cancer patients.

We found that women with physical and financial barriers, psychological barriers, and a lack of proper knowledge were more likely to experience delays in diagnosis and treatment. One plausible explanation for these positive associations was that a majority of our study participants resided in rural areas, had lower incomes, and lower educational attainment. Thus, the cost of medical care services, travel time to the hospital, distance from patients’ homes to the hospital, family and work responsibilities, inadequate awareness, behaviors, and fears could be major obstacles for accessing medical care. This is supported by the results of our stratified analysis. Some evidence from developed countries indicates that women with lower incomes show a higher risk of delay, while lower educational attainment levels create barriers, which contribute to a delay in seeking a medical appointment.^21^, ^22^ Furthermore, Vietnamese women residing in rural areas are more likely to use complementary and herbal or alternative medical treatment at the appearance of first symptoms, which was found to be associated with a delay in diagnosis and treatment of breast cancer.^23^

In our study, we found that women who lacked proper knowledge were more likely to experience delays in diagnosis and treatment, particularly young women (<45 years old) and those with high incomes. There may be a misconception that breast cancer is a disease of older women. Some breast cancer‐related symptoms, such as pain, tenderness, and lumps, are non‐cancer specific. Therefore, young women and their health consultants/professionals may not consider breast cancer as a possible diagnosis.^24^ One study also found that “being too busy to bother with their concerns” was more frequently reported in high socioeconomic groups, which may explain our findings for high‐income women.^25^ Regardless, our findings suggest the importance and necessity of increased education and awareness of breast cancer among young women and their health care providers.

There are no national breast cancer screening guidelines or programs implemented in Vietnam. Although breast cancer screening is available in center/tertiary hospitals and some private hospitals, screening procedures are not covered by the national health insurance system. The lack of guidelines and insurance coverage for breast cancer screening may explain why the majority of patients in our study were diagnosed when symptoms presented.

One limitation of our study is that we were not able to separate patient delay time and health system delay time. A health system delay is typically defined as a delay of at least 1 month within the health care system from the first medical visit to the initiation of cancer treatment. About two‐thirds of our study participants reported immediately seeking medical care after the recognition of first symptoms, and the median time for cancer diagnosis and treatment was 2.0 months for these patients. This may be considered as a proxy of the median health system delay time. Currently, there are five comprehensive cancer centers and 70 oncology departments located within center/tertiary and provincial hospitals throughout Vietnam.^26^ The Vietnam National Cancer Hospital and Hanoi Oncology Hospital are two of five comprehensive cancer centers. Cancer patients in Vietnam often seek medical care at center/tertiary hospitals after the recognition of first symptoms or are referred to cancer centers or tertiary hospitals for diagnosis confirmation. Cancer centers and tertiary hospitals are overcrowded and overwhelmed, resulting in increasing numbers of breast cancer patients seeking cancer diagnosis and treatment at private hospitals. Nevertheless, most breast cancer patients in Vietnam are still diagnosed and treated at cancer centers, tertiary, and provincial hospitals.^27^ Residents of Hanoi and its surrounding area have more opportunities to access health care and receive more timely diagnoses and treatment than women living in smaller cities. However, results of our study suggest that health system delays exist in Vietnam, even in the capital city of Vietnam, from where all our study participants were recruited. In participants who reported postponements in seeking medical care, the median total delay time was 5.5 months. This suggests that the patient delay time may account for a major portion of the total delay time. While patient delay time most likely reflects patients’ poor knowledge, perception, cultural beliefs, and barriers,^28^ studies have indicated that health system delay time most likely reflects access barriers and quality deficiencies in cancer care throughout LMIC health systems.^29^ Studies are needed to directly access health system delays and their contributions to breast cancer treatment in Vietnam.

It should be noted that, although the response rate was high (93.1%) for patients approached by our research team, our study only included ~35% of newly diagnosed breast cancer patients seeking care at the Vietnam National Cancer Hospital and Hanoi Oncology Hospital from July 2017 to June 2018. Thus, selection bias cannot be completely ruled out. Because information pertaining to reasons for delays in seeking medical care was collected within 2–3 weeks from hospital admission and through a structured questionnaire administered by trained interviewers, recall bias and exposure misclassifications were minimized. However, information bias is inevitable when a self‐report is the only source. Furthermore, the validity and reliability of our survey questionnaire have not been evaluated in a Vietnamese population. Interpretation of our study results should keep these limitations in mind. Finally, our findings may be not generalizable to breast cancer patients living in other parts of Vietnam.

In conclusion, substantial diagnosis and treatment delays exist among female breast cancer patients in Vietnam. Patients who had financial and physical barriers, psychological barriers, or a lack of proper knowledge were more likely to experience delays in diagnosis and treatment. Our study highlights the need to develop a proper public health policy to address these concerns in Vietnam, particularly for women living in rural areas or older women. More research is needed to quantify access barriers and quality deficiencies in breast cancer care in Vietnam.

## ETHICS STATEMENT

Written informed consent was obtained from all VBCS participants. Ethical approvals for human subject research were obtained from the Vietnam National Cancer Institute and Vanderbilt University Medical Center.

## CONFLICT OF INTERESTS

The author(s) declared no potential conflicts of interest with respect to the research, authorship, and/or publication of this article.

## Supporting information

Supplementary Material

## Data Availability

Data are available on request. The data underlying this article will be shared at reasonable request to the corresponding author.
