# Supplementary material for: Delay in the diagnosis and treatment of breast cancer in Vietnam
Source: Cancer Med. 2021 Oct 19;10(21):7683–91. doi: 10.1002/cam4.4244 (PMC8559509; doi:10.1002/cam4.4244)
Supplement: Supplementary file 1 — Supplementary Material [file CAM4-10-7683-s001.docx]

**538** newly diagnosed with breast cancer patients

at National Cancer hospital and Hanoi Oncology Hospital (7/2017 – 6/2018)

Patients refused (n = 37)

**501** patients (21-75 years of age) agreed to participate and completed a baseline survey (Participation rate: 93%)

Excluded participants have a confirmatory diagnosis as a benign tumor based on Medical chart review (n = 9)

**492** participants have a confirmatory diagnosis as a malignin tumor from clinical and pathological reports

**472** Cases completed medical chart review (12/2019-5/2020)

**Finally, 462** cases remained

Patients uncompleted a medical chart review (n = 20)

Patients as non-symptomatic breast cancer (n = 10)

**Supplemental Figure 1:** Flowchart of participant selections for the analysis

**Supplemental Table 1:** **Clinical features of breast cancer patients (N=462)**

| **Clinical features** | **n (%)** |
| --- | --- |
| **Molecular subtypes** |  |
| Luminal A | 74 (16.0) |
| Luminal B – HER2/neu negative | 109 (23.6) |
| Luminal B – HER2/neu positive | 102 (22.1) |
| HER2/neu overexpression | 92 (19.9) |
| Basal subtype/ triple negative breast cancer | 46 (10.0) |
| ER-negative and PR-positive | 19 (4.1) |
| Unavailable | 20 (4.3) |
| **Tumor (T) stage** |  |
| Tis | 2 (0.4) |
| 1 | 116 (25.1) |
| 2 | 255 (55.9) |
| 3 | 35 (7.6) |
| 4 | 35 (7.6) |
| Unavailable | 19 (4.1) |
| **Node (N) stage** |  |
| 0 | 250 (54.1) |
| 1 | 118 (25.5) |
| 2 | 67 (14.5) |
| 3 | 18 (3.9) |
| Unavailable | 9 (2.0) |
| **Metastasis (M) stage** |  |
| 0 | 434 (93.9) |
| 1 | 22 (4.8) |
| Unavailable | 6 (1.3) |
| **Stage at diagnosis (AJCC 8^th^ edition)** |  |
| 0 | 2 (0.4) |
| I | 91 (19.7) |
| 2A | 160 (34.6) |
| 2B | 76 (16.5) |
| 3A | 59 (12.8) |
| 3B | 24 (5.2) |
| 3C | 12 (2.6) |
| 4 | 22 (4.8) |
| Unavailable | 16 (3.5) |

*AJCC: American Joint Committee on Cancer*

**Supplemental Table 2: Participant’s demographics, diagnosis modality, the first noticed symptoms and barriers for delay in seeking medical care and by the delay in diagnosis and treatment.**

|  | **Delay in diagnosis and treatment** | | | *P value* |
| --- | --- | --- | --- | --- |
|  | No delay | Moderate delay | Serious delay |  |
|  | N=238 | N=143 | N=81 |  |
| **Age at diagnosis (Mean±SD;** years old**)** | 49.7±11.5 | 48.9±10.3 | 49.7±8.3 |  |
| < 45 | 74 (31.1) | 49 (34.3) | 24 (29.6) | 0.71 |
| 45-54 | 91 (38.2) | 50 (35.0) | 34 (42.0) |  |
| 55-64 | 57 (24.0) | 33 (23.1) | 21 (5.9) |  |
| 65+ | 16 (6.7) | 11 (7.7) | 2 (2.5) |  |
| **Marital Status** |  |  |  |  |
| Married | 201 (84.5) | 118 (82.5) | 69 (85.2) | 0.84 |
| Single/ separated/ divorced/ windowed | 37 (15.5) | 25 (17.5) | 12 (14.8) |  |
| **Education** |  |  |  |  |
| Never had formal education/ primary school | 38 (16.0) | 20 (13.9) | 12 (14.8) | 0.50 |
| Middle school | 101 (42.4) | 71 (49.7) | 32 (39.5) |  |
| High school | 51 (21.4) | 31 (21.7) | 24 (29.6) |  |
| College or higher | 48 (20.2) | 21 (14.7) | 13 (16.1) |  |
| **Occupation** |  |  |  |  |
| Workers in agriculture/ in industrial and construction | 109 (45.8) | 74 (51.8) | 39 (48.2) | 0.78 |
| Governors/managers/officers | 55 (23.1) | 26 (18.2) | 18 (22.2) |  |
| Servicers/Sellers/homemakers/students and others | 74 (31.1) | 43 (30.1) | 24 (29.6) |  |
| **Average annual per capita income** (Mean±SD**;** million VND**)** | 23.5±15.9 | 23.2±16.9 | 19.1±13.3 | *0.04* |
| **Location** |  |  |  |  |
| Urban/ Sub-urban area | 102 (42.9) | 49 (34.3) | 27 (33.3) | 0.14 |
| Rural area | 136 (57.1) | 94 (65.7) | 54 (66.7) |  |
| **Travel time to a health care setting** (minutes) | |  |  |  |
| < 30 | 76 (31.9) | 42 (29.4) | 19 (23.4) | 0.78 |
| 30 | 47 (19.8) | 34 (23.8) | 20 (24.7) |  |
| 31-60 | 48 (20.2) | 30 (21.0) | 20 (24.7) |  |
| > 60 | 67 (28.2) | 37 (25.9) | 22 (27.2) |  |
| **Family history** |  |  |  |  |
| Breast cancer-first degree | 9 (3.8) | 4 (2.8) | 4 (4.9) | 0.71 |
| Other cancers-first degree | 45 (18.9) | 19 (13.3) | 17 (21.0) | 0.25 |
| **The number of symptoms noticed** | |  |  |  |
| Only one | 145 (60.9) | 73 (51.1) | 32 (39.5) | *0.004* |
| Two | 66 (27.7) | 51 (35.7) | 29 (35.8) |  |
| Above two | 27 (11.3) | 19 (13.3) | 20 (24.7) |  |
| **Circumstance of breast cancer detection** |  |  |  |  |
| Mammographic screening | 2 (0.8) | 3 (2.1) | 0 (0) | 0.51 |
| Routine health examination | 15 (6.3) | 7 (4.9) | 3 (3.7) |  |
| Self-noticed systems/lump | 220 (92.8) | 133 (93.0) | 78 (96.3) |  |
| **Barriers for delay in seeking a medical care** | |  |  |  |
| Financial and physical barriers | 17 (7.1) | 43 (30.1) | 20 (24.7) | *<0.001* |
| Psychological barriers | 4 (1.7) | 14 (9.8) | 5 (6.2) | *0.001* |
| A lack of proper knowledge | 12 (5.0) | 21 (14.7) | 9 (11.1) | *0.004* |
